# *EGFR*突变丰度的检测及其临床意义

**DOI:** 10.3779/j.issn.1009-3419.2017.08.11

**Published:** 2017-08-20

**Authors:** 宜 邵, 殿胜 钟

**Affiliations:** 300052 天津，天津医科大学总医院肿瘤内科 Department of Medical Oncology, Tianjin Medical University General Hospital, Tianjin 300052, China

**Keywords:** 肺肿瘤, 表皮生长因子受体, 丰度, 酪氨酸激酶抑制剂, Lung neoplasms, Epidermal growth factor receptor, Abundance, Tyrosine kinase inhibitors

## Abstract

具有表皮生长因子受体（epidermal growth factor receptor, *EGFR*）敏感突变的非小细胞肺癌患者对酪氨酸激酶抑制剂（tyrosine kinase inhibitors, TKIs）具有良好反应，但是即使存在相同突变，不同患者对TKIs的反应也不一致。推测突变分子含量的不同是TKIs反应不一的部分原因，具有更高*EGFR*突变“丰度”的肿瘤患者可能从TKIs中获益更多。*EGFR*突变可根据检测方法敏感性的不同，定量检测突变分子比例以及突变蛋白表达等进行半定量或定量检测。*EGFR*突变丰度可能有助于反映肿瘤异质性，评估疾病进程，预测TKIs药物敏感性，早期发现耐药，具有重要的临床意义。

## Abstract (en)

Non-small cell lung cancer (NSCLC) patients, with sensitive epidermal growth factor receptor (*EGFR*) mutations react well to tyrosine kinase inhibitors (TKIs). However, the efficacy of TKIs on patients with the same mutant types differs dramatically. It is implied that the different quantities of mutant alleles could be one of the reasons underlying. Patients with high abundance of *EGFR* mutation might benefit more from TKIs. There are no universal standards for the definition of *EGFR* mutant abundance. Abundance could be semi-quantified according to the different sensitivities of detection methods, quantified with quantifying detection techniques such as digital PCR or next generation sequencing, or quantified based on the expression of mutant proteins. The different abundances of primary and metastatic diseases could reflect the heterogeneity of the tumors. The pre-treatment level or the dynamic change of *EGFR* mutant abundance could help observe the course of the diseases and predict the efficacy of TKIs. TKIs resistance could be detected by change of abundance prior to image manifestations. Besides, the abundance of T790M could also predict drug efficacy and resistance of the first and third generation TKIs. Thus the detection of *EGFR* mutant abundance has important clinical significance. The standardization and correction of abundance needs more exploration.

肺癌是世界上最常见的恶性肿瘤之一，其中非小细胞肺癌（non-small cell lung cancer, NSCLC）约占肺癌的80%^[1]^。大多数NSCLC在诊断时即处于进展期，对于具有敏感突变基因的患者来说，靶向治疗为其标准治疗。多项研究^[2, 3]^证实具有表皮生长因子受体（epidermal growth factor receptor, *EGFR*）敏感突变的患者对酪氨酸激酶抑制剂（tyrosine kinase inhibitors, TKIs）具有良好反应，总反应率为55%-80%，无进展生存期（progression-free survival, PFS）为9个月-14个月。但是，即使具有相同类型乃至相同亚型，不同患者对TKIs的反应也不一致。因此，预测患者从TKIs治疗中的获益程度具有重要意义。

之前研究发现，肿瘤组织的分子异常存在异质性，即同一肿瘤中同时含有*EGFR*突变和野生型克隆^[4]^，因此推测突变分子含量的不同是TKIs反应不一的部分原因，具有更高*EGFR*突变“丰度”的肿瘤患者可能从TKIs中获益更多，仅仅定性分析突变来指导靶向治疗已不能满足临床需求。随着数字PCR（digital PCR, dPCR）、二代测序（next generation sequencing, NGS）等方法的出现，定量检测*EGFR*突变已经成为可能。但是，*EGFR*突变丰度尚无统一定义，其与突变型肺癌及TKIs疗效的关系也并无定论。本文将目前*EGFR*突变丰度的含义及其临床意义综述如下。

## 1 *EGFR*突变丰度的定义及检测方法

丰度（abundance）概念来自于化学，最初指元素的相对含量。而肿瘤学中所谓*EGFR*基因突变的分子丰度还没有统一的定义和标准，一般指突变型*EGFR*突变基因相对或绝对定量值，丰度的概念也随检测方法和检测样本的不同而各有不同。

### 1.1 *EGFR*突变丰度的半定量检测

早期的研究根据突变检测方法敏感性的不同来定义丰度。一般来说，*EGFR*突变频率需达到20%-30%才能由直接测序发现，而扩增阻滞突变系统（amplification refractory mutation system, ARMS）可检测到1%的突变^[5]^。根据二者敏感性的差别，2010年Zhou等^[6]^使用直接DNA测序和ARMS法检测100例肺癌样本，将两种方法都为阳性者定义为*EGFR*突变高丰度，ARMS法阳性、测序阴性为低丰度，两种方法都为阴性者为野生型，并发现高低丰度者对TKIs反应确有不同。此研究虽然具有重要的临床意义，但仅用两种方法的敏感性差异来判断突变丰度较为粗糙，本质上仍属于半定量方法。此外，尽管研究要求样本的肿瘤细胞比例 > 50%，但一定比例的正常细胞仍有可能干扰检测结果，特别是对直接测序的影响较大。之后同样有研究使用类似方法重复了此研究，但并未发现此种丰度和TKI反应之间具有相关性^[7]^。

### 1.2 *EGFR*突变丰度的定量检测

随着突变定量检测方法的出现，更多研究使用*EGFR*突变和野生型拷贝的比值来定义丰度。2009年，Yung等^[8]^第一次使用丰度概念并用dPCR将*EGFR*突变定量化。研究使用微滴数字PCR（droplet digital PCR, ddPCR）方法来定量NSCLC患者血浆和肿瘤组织的*EGFR*常见突变（19外显子缺失和L858R点突变）。ddPCR敏感性高，可检测到临床样本的单个突变DNA分子，并准确测定突变及野生序列的数量。作者发现检测到的比例浓度可低至0.1%，并将丰度定义为突变分子数在总的DNA中所占浓度。研究发现突变患者接受TKI治疗后出现*EGFR*突变丰度的下降，但没有分析基线突变丰度和疗效预测及预后价值。

Wang等^[9]^使用纳流控dPCR分析平台（nanofluidic digital PCR assay platform），通过计算*EGFR*分子和单拷贝基因RPP30数量的比值来确定基因组DNA拷贝中含有多少突变分子，也即*EGFR*基因拷贝的相对数。此种方法检测下限可低至0.02%，检测到20个手术切除样本的*EGFR*突变丰度为0.02%-9.26%。

Marchetti等^[10]^使用半定量指数（semiquantitative index, SQI）定量*EGFR*突变。SQI来自包含突变*EGFR*和固定量野生型*EGFR*的已知拷贝数稀释系列，并以qPCR中野生型DNA做为内参，反映*EGFR*突变基因对比野生型拷贝数的趋势。研究检测了69例*EGFR*突变肿瘤和21例阴性对照患者的血浆，发现SQI和厄洛替尼治疗后反应相关。

Yang等^[11]^的研究使用ddPCR方法定量和动态检测循环肿瘤DNA（circulating tumor DNA, ctDNA）的*EGFR*突变，发现ddPCR检测的下限是0.04%。以肿瘤组织的*EGFR*突变为金标准，血浆ctDNA的一致率为74%（54/73）。ddPCR检测循环*EGFR*突变等位基因和野生型等位基因的绝对量，突变*EGFR* ctDNA和野生型ctDNA的浓度比值定义为*EGFR*突变丰度。其中，54例未治疗突变患者血浆中中位绝对和相对*EGFR*突变等位基因量是487拷贝/反应和5.15%，因此，研究将相对*EGFR*突变量 > 5.15%者定义为高丰度（*n*=27），而≤5.15%者为低丰度（*n*=27）。

关于厄洛替尼和化疗间插治疗的FASTACT-2研究^[12]^前瞻性用Cobas检测基线、第3周期和疾病进展（progressive disease, PD）的血液标本，检测血浆游离DNA（cell-free DNA, cfDNA）*EGFR*突变状态，并使用突变拷贝数/血浆毫升数定量监测TKIs治疗过程中动态变化。但此丰度定义方法可能容易受到非肿瘤DNA数量等影响，是否准确还需要进一步验证。

NGS同样可用来检测突变丰度。2016年，Zhang等^[13]^使用单分子扩增和再测序技术（single-molecule amplification and resequencing technology, SMART）行基因检测，将突变率定义为突变等位基因数量/总等位基因数量。以ARMS做为标准，SMART的敏感性和特异性分别是98.7%和99.0%。但研究并未发现丰度和使用TKIs的PFS之间具有相关性。

由于对一代TKIs耐药机制的深入理解和三代TKIs的出现，T790M定量检测的重要性也逐渐被认识到。有研究^[14]^将T790M等位基因数量对比活化突变等位基因数量进行T790M相对定量，使用磁珠乳液扩增PCR（bead, emulsion, amplification, magnetic, BEAMing）检测23例TKIs后PD患者和21例突变未经治患者的ctDNA，发现T790M比活化突变值为13.3%-94.0%。检出率在PD患者中更高，两组分别是82.6%和61.9%（*P*=0.18）。还有研究使用相同定量方法检测T790M及活化突变在TKIs治疗前后变化^[15]^，使用dPCR检测TKIs治疗前后肿瘤组织。三代TKIs药物rociletinib的Ⅰ期/Ⅱ期研究对PD患者进行活检，同样检测T790M对活化突变等位基因的相对频率^[16]^。

有研究使用dPCR检测cfDNA *EGFR* T790M，以突变T790M/（突变T790M+野生型等位基因）比值进行定量，并以5%和0为界进行高/低/无分组，研究其基线值和TKIs反应关系，及动态变化和疗效关系^[17]^。同样地，上文^[13]^提到的SMART技术也可定量检测T790突变，将突变率定义为突变等位基因数量/总等位基因数量，用ARMS和SMART测得T790M突变率分别是1.1%和2.2%。三代TKI奥希替尼的临床研究同样使用NGS来检测T790M丰度和疗效的相关性^[18]^。

### 1.3 蛋白检测

由于DNA测序的敏感性依赖于肿瘤细胞在样本中的比例，特别是依赖于携带突变的肿瘤细胞百分比。因此，有研究^[19]^发现针对两个最常见*EGFR*突变的特异性兔单克隆抗体，用Western blot、免疫荧光、免疫组化（immunohistochemistry, IHC）检测340例NSCLC患者的组织突变，和直接及以质谱为基础的DNA测序方法相比，敏感性为92%，特异性为99%。不同于DNA测序，IHC的结果依赖于单个肿瘤细胞染色的强度，而不是整个肿瘤裂解物。因此，肿瘤样本突变如果有低百分比的*EGFR*突变肿瘤细胞，DNA测序可能难以测出，但能被IHC突变特异性抗体检测到，尤其是在小样本标本具有优势。此外，IHC也较少受到癌细胞空间分布的影响。但抗体只针对*EGFR*的特定常见突变，其他突变无法测出。

之后有研究^[20]^使用IHC定量检测47例PCR证实突变的NSCLC患者，表达分数由染色强度和表达突变*EGFR*肿瘤组织的比例共同决定。表达*EGFR*突变的肿瘤细胞比例（比例分数）：0：无；1分：1%-10%；2分：11%-30%；3分：31%-50%；4分：51%-70%；5分：71%-100%。染色强度（强度分数）： > 10%的肿瘤细胞中，0：不染色；1分：弱染色；2分：中度染色；3分：强染色。总表达分数为二者相加。19外显子缺失和L858R突变的中位分数分别是4分和7分，因此将19外显子缺失定量为：0分-3分：低表达，4分-8分：高表达；L858R定量为：0分-6分：低表达，7分-8分：高表达。

## 2 *EGFR*突变丰度的临床意义

### 2.1 反映肿瘤异质性

有研究^[21]^使用qPCR检测突变比。如上文定量方法所述，突变比=突变水平/（突变水平+野生等位基因水平）。检测50例NSCLC患者的原发灶和转移灶，原发灶不同部位的定量平均突变偏差是18.3%（范围0-54.3%），具有高水平一致性。而转移灶肿瘤*EGFR*突变比显著低于原发灶，Kappa值是0.615（*P* < 0.01），这一研究说明尽管为同一来源肿瘤，原发灶和转移灶的突变丰度也具有异质性，也可以解释在TKIs治疗过程中，不同病灶反应不一。

### 2.2 突变丰度与疾病转归

Yung的研究中^[8]^，突变序列的血浆浓度和临床反应一致性良好。所有部分缓解（partial remission, PR）和完全缓解（complete remission, CR）的患者浓度降低，而PD患者突变持续。其中一个患者初始反应后因药物不良反应停用TKI，停药后4周肿瘤相关*EGFR*突变再次出现，证明*EGFR*丰度变化可反映疾病动态变化，但研究未阐述基线丰度水平和疗效之间的关系。

TKIs治疗过程中血浆*EGFR*突变动态定量改变和临床结局的关系还不清楚。Ⅲ期随机对照研究CTONG0901^[22]^探索性分析了血浆*EGFR* L858R突变和TKIs反应及耐药的关系。使用qPCR检测80例患者血浆的相对L858R拷贝数，发现在对TKI反应最好时L858R量最低。和基础L858R量相比，进展时量增加超过一倍作为分界，61例患者进展时L858R增长到最高水平（上升型），19例患者进展时稳定不变（稳定型），上升型的PFS长于稳定性，两组的中位PFS分别是11.1个月和7.5个月。但此种方法为回溯性分析，缺乏疗效预测价值。

### 2.3 突变丰度预测TKIs疗效

初治患者的基线*EGFR*丰度似可预测TKIs疗效。Zhou等^[6]^的半定量丰度研究中，100个月接受检测的患者中51个月为高丰度，18个月为低丰度，接受吉非替尼治疗后，PFS分别是11.3个月和6.9个月（*P*=0.014），说明基线的相对*EGFR*突变丰度可以预测TKI获益程度。但Chiu等^[7]^发现高低丰度患者对TKI反应没有显著不同，直接测序和ARMS法检测突变结果不同和相同患者的PFS分别是13.4和10.9个月，没有显著统计学差异。但Zhou的研究和Chiu的研究不同之处在于，前者只纳入肿瘤细胞超过50%的样本，而后者大约60%的标本肿瘤细胞小于50%，不能除外肿瘤细胞比例对直接测序突变检测的影响。

吉非替尼治疗患者使用IHC进行*EGFR*突变定量，高分比低分的PFS显著延长，分别是12.2个月和3.4个月（*P* < 0.001），证实肿瘤组织*EGFR*突变表达的定量分析可预测TKIs疗效。

同样，丰度动态变化也可反映TKIs疗效。血浆*EGFR*突变快速降低预示更好的TKIs反应^[10]^。FASTACT-2研究，*EGFR*突变特异性cfDNA水平在第3周期降低，进展时升高。第3周期时血浆*EGFR*突变依旧阳性预示较差的临床结局^[12]^。

Yang ^[11]^的研究发现，同为肿瘤组织突变阳性患者，和血浆野生型（*n*=19）相比，ctDNA突变的患者（*n*=54）具有更好的PFS（12.6个月*vs* 6.7个月，*P* < 0.001）和OS（35.6个月*vs* 23.8个月，*P*=0.028）。和低*EGFR*丰度（≤5.15%）患者相比，高*EGFR*突变丰度患者（> 5.15%）具有更好的PFS（15.4个月*vs* 11.1个月，*P*=0.021）。分析67例进展后患者血浆DNA，43.3%患者出现丰度降低，19.4%不变，37.3%升高。降低组具有更好的PFS（12.7个月*vs* 7.1个月，*P*=0.001）和OS（28.3个月*vs* 14.9个月，*P*=0.027）。T790M的出现和丰度变化趋势无关。

FASTACT-2研究中^[12]^，总的*EGFR*突变特异性cfDNA水平在第3周期降低，在PD时回归。和化疗+安慰剂组对比，在第3周期和PD时化疗+厄洛替尼治疗组突变*EGFR*等位基因数量更少（第3周期：5拷贝/mL *vs* 0；PD：83拷贝/mL *vs* 6拷贝/mL）。厄洛替尼组第3周期仍可检测到突变*EGFR*等位基因的患者PD风险增加62%，死亡风险增加55%，因此，第3周期时的cfDNA *EGFR*突变阳性是更差PFS和OS的预测因子。

另有研究^[23]^分析9例TKIs PD患者，所有患者接受厄洛替尼治疗后都出现血浆*EGFR*突变丰度降低，其中8例患者达到血浆CR，1例没有出现血浆CR的患者则出现了早期进展。6例患者在客观进展时血浆中又检测到了突变*EGFR*，血浆PD出现在在RECIST进展前4周-12周。

使用SQI半定量检测血浆*EGFR*突变^[10]^，TKIs治疗后的4 d-60 d系列*EGFR*检测出现进行性SQI下降。经过TKIs4 d治疗后，SQI平均降低13.5%，8 d降低41.6%，14 d降低63.5%。除了2例患者，在8 d-60 d后血浆检测都为阴性。70%患者（快速反应者）在14 d时下降超过50%，另外30%的患者（缓慢反应者）在14 d时下降小于50%。2个月时SQI的降低率和肿瘤缩小比例（percentage of tumor shrinkage, PTS）显著相关，快速反应者PTS平均降低（59.1±1.8），缓慢反应者则降低（18.3±3.7）（*P* < 0.000, 1）。2例慢反应者*EGFR*突变没有完全清除，在35 d时T790M就意外出现，之后进一步增长，这些病例被定义为早期耐药。因此，连续检测血浆基因型可能是反映靶向治疗疗效的良好临床分子标志，也是潜在的早期临床试验终点。

有研究^[24]^探索了突变丰度和再使用TKIs的关系。检测51例TKIs获得性耐药患者，使用qPCR检测突变，*EGFR*突变丰度=突变*EGFR*量/（突变量+野生量）。51例突变NSCLC患者的中位丰度是0.501, 5，因此将丰度≥0.501, 5高丰度（H）， < 0.501, 5为低丰度（L）。和L组相比（27/51），H组患者（24/51）再次使用一代TKIs具有显著延长的PFS（5.27个月*vs* 2.53个月，*P*=0.033）。因此，使用一代TKIs耐药后，具有更高突变*EGFR*丰度是接受TKIs再治疗的潜在指标，可能是因为依赖于*EGFR*信号通路的肿瘤细胞比例更高。

### 2.4 T790M突变丰度的临床意义

T790M突变丰度的增加可能和一代TKIs耐药具有相关性。有研究^[15]^使用dPCR检测到所有25个组织中T790M（治疗前13个样本，治疗后12个样本），计算活化突变（L858R）或T790M等位基因和对照等位基因数量比值。用药前L858R/对照=65.62%，T790M/对照=0.34%，T790M等位基因和活化突变等位基因数量比（T/A）=0.52%。用药后L858R/对照=3.87%，T790M/对照=1.89%，T/A=48.84%。10例低T/A比值，TKIs治疗显著肿瘤退缩。其中6例耐药患者T/A出现显著增加。因此，一代TKIs治疗过程中，T790M丰度的增加可能提示出现获得性耐药。

但也有研究得出不同结论。有研究用dPCR检测TKIs治疗前后血浆cf-DNA T790M的变化^[17]^，发现TKIs治疗前T790M阳性患者具有更差的PFS（8.9个月*vs* 12.1个月，*P*=0.007）和OS（19.3个月*vs* 31.9个月，*P*=0.001），而基线高丰度（> 5%）T790M预示着更差的PFS（7.1个月*vs* 9.5个月，*P*=0.001）。但在TKIs治疗过程中，T790M数量增加者反而具有更优PFS（11.6个月*vs* 7.1个月，*P*=0.044）和OS（26.3个月*vs* 19.3个月，*P*=0.015）。是否天然和获得性T790M的临床意义不同还需要进一步探索。

治疗前T790M阳性细胞比例可预示对T790M阳性肿瘤对三代TKIs rociletinib的治疗反应。25例接受rociletinib治疗的患者，基线T790M阳性细胞比例更高者，rociletinib治疗后具有更大的肿瘤退缩，说明T790M负荷定量信息可提示患者治疗反应，肿瘤中定量等位基因频率是预测最大获益的指标^[15]^。同样，奥希替尼的Ⅰ期AURA研究的事后分析以相对等位基因比例（allelic fraction, AF），也即*EGFR*突变cfDNA和野生型*EGFR* cfDNA的比值进行突变定量，发现cfDNA T790M突变阳性患者，组织T790M阳性肿瘤（*n*=108）的AF值高于T790M阴性肿瘤（*n*=16）（相对T790M AF，33.6% *vs* 16.8%，*P*=0.004, 7）。相对T790M AF增加和最大反应深度并不总相关（R=-0.183），但和AF < 10%患者相比，相对T790M AF > 10%的患者具有更大的反应深度（*P*=0.040, 7）。但一项关于奥希替尼治疗血检T790M阳性患者的临床试验中^[18]^，较小的T790M突变等位基因比例和更大的肿瘤退缩具有相关趋势，反应最好的7例患者中（肿瘤退缩大于50%），3例T790M < 0.25%，似乎提示任何丰度的T790M都可从三代TKIs中获益。

T790M的动态变化可能和三代TKIs疗效相关。TIGER-X研究中^[25]^，NGS检测患者ctDNA T790M突变拷贝数的绝对值，发现三代TKIs rociletinib治疗后，血检*EGFR*突变水平下降可能可预测反应深度。检测9例患者尿液，发现不论最佳确认反应为何，第1周期对比基线都出现尿T790M水平的显著下降（范围-51%到-100%；*P*=0.009, 1）。但是在2例PD患者，下降受阻（-51%和-70%），而PR或SD为最佳反应的患者下降范围为-83%到-100%，反映了rociletinib可降低T790M阳性克隆增殖。

## 3 总结

dPCR、NGS等方法的出现使*EGFR*定量检测成为可能。尽管临床还没有标准定义及检测*EGFR*突变的方法，但*EGFR*突变丰度的检测及其意义已经日益引起人们的重视。不同病灶的不同丰度可以反映肿瘤异质性，*EGFR*活化突变的基线丰度值可能和TKIs疗效相关，TKIs治疗过程中*EGFR*丰度的变化对于检测疗效、早期发现进展等具有重要意义。T790M耐药突变丰度的增加可能可早于临床提示一代TKIs获得性耐药，其变化也是三代TKIs疗效的良好预测因子。总之，*EGFR*突变丰度检测具有十分重要的临床意义。

但是当前对突变丰度的应用还存在明显不足。丰度概念混杂，没有统一标准，各种检测方法缺乏绝对可比性。另外，突变拷贝数的检测在一定程度上受到各种标本所含肿瘤细胞的比例不同的影响，而且很多*EGFR*突变肺癌合并存在*EGFR*基因扩增，丰度检测是否需要进行肿瘤细胞比例和基因扩增的校正还需要更多的探索。
